# Natural Killer Cells: The Linchpin for Successful Cancer Immunotherapy

**DOI:** 10.3389/fimmu.2021.679117

**Published:** 2021-04-28

**Authors:** Kari A. Shaver, Tayler J. Croom-Perez, Alicja J. Copik

**Affiliations:** ^1^ College of Medicine, University of Central Florida, Orlando, FL, United States; ^2^ Burnett School of Biomedical Science, College of Medicine, University of Central Florida, Orlando, FL, United States

**Keywords:** natural killer (NK) cells, NK cells and immunotherapy, NK cells and checkpoint blockade, NK cell crosstalk, immunotherapy resistance, adoptive NK cell therapy, immuno-oncology combinations, NK cell dysfunction

## Abstract

Cancer immunotherapy is a highly successful and rapidly evolving treatment modality that works by augmenting the body’s own immune system. While various immune stimulation strategies such as PD-1/PD-L1 or CTLA-4 checkpoint blockade result in robust responses, even in patients with advanced cancers, the overall response rate is low. While immune checkpoint inhibitors are known to enhance cytotoxic T cells’ antitumor response, current evidence suggests that immune responses independent of cytotoxic T cells, such as Natural Killer (NK) cells, play crucial role in the efficacy of immunotherapeutic interventions. NK cells hold a distinct role in potentiating the innate immune response and activating the adaptive immune system. This review highlights the importance of the early actions of the NK cell response and the pivotal role NK cells hold in priming the immune system and setting the stage for successful response to cancer immunotherapy. Yet, in many patients the NK cell compartment is compromised thus lowering the chances of successful outcomes of many immunotherapies. An overview of mechanisms that can drive NK cell dysfunction and hinder immunotherapy success is provided. Rather than relying on the likely dysfunctional endogenous NK cells to work with immunotherapies, adoptive allogeneic NK cell therapies provide a viable solution to increase response to immunotherapies. This review highlights the advances made in development of NK cell therapeutics for clinical application with evidence supporting their combinatorial application with other immune-oncology approaches to improve outcomes of immunotherapies.

## Introduction

Cancer immunotherapy is a rapidly evolving treatment modality that works by augmenting the body’s own immune system. The dramatic successes of cancer immunotherapies have led to a paradigm shift in oncology (1, 2). While various immune stimulation strategies such as checkpoint blockade of PD-1/PD-L1 or CTLA-4 have been a major step forward leading to durable responses even in patients with advanced cancers, the overall response rate is low. Responses to anti-PD-1/PD-L1 therapies were shown to correlate with expression of PD-L1 on tumors and with preexistence of inflamed (“hot”) tumors infiltrated with functional cytotoxic lymphocytes, which accounts for a minority of patients (3, 4). While the application of immune checkpoint inhibitors is known to mount the potent antitumor response by cytotoxic T cells, there is a robust body of science suggesting that immune responses independent of cytotoxic T cells also play critical roles in the efficacy of immunotherapeutic interventions.

**Figure 1:**
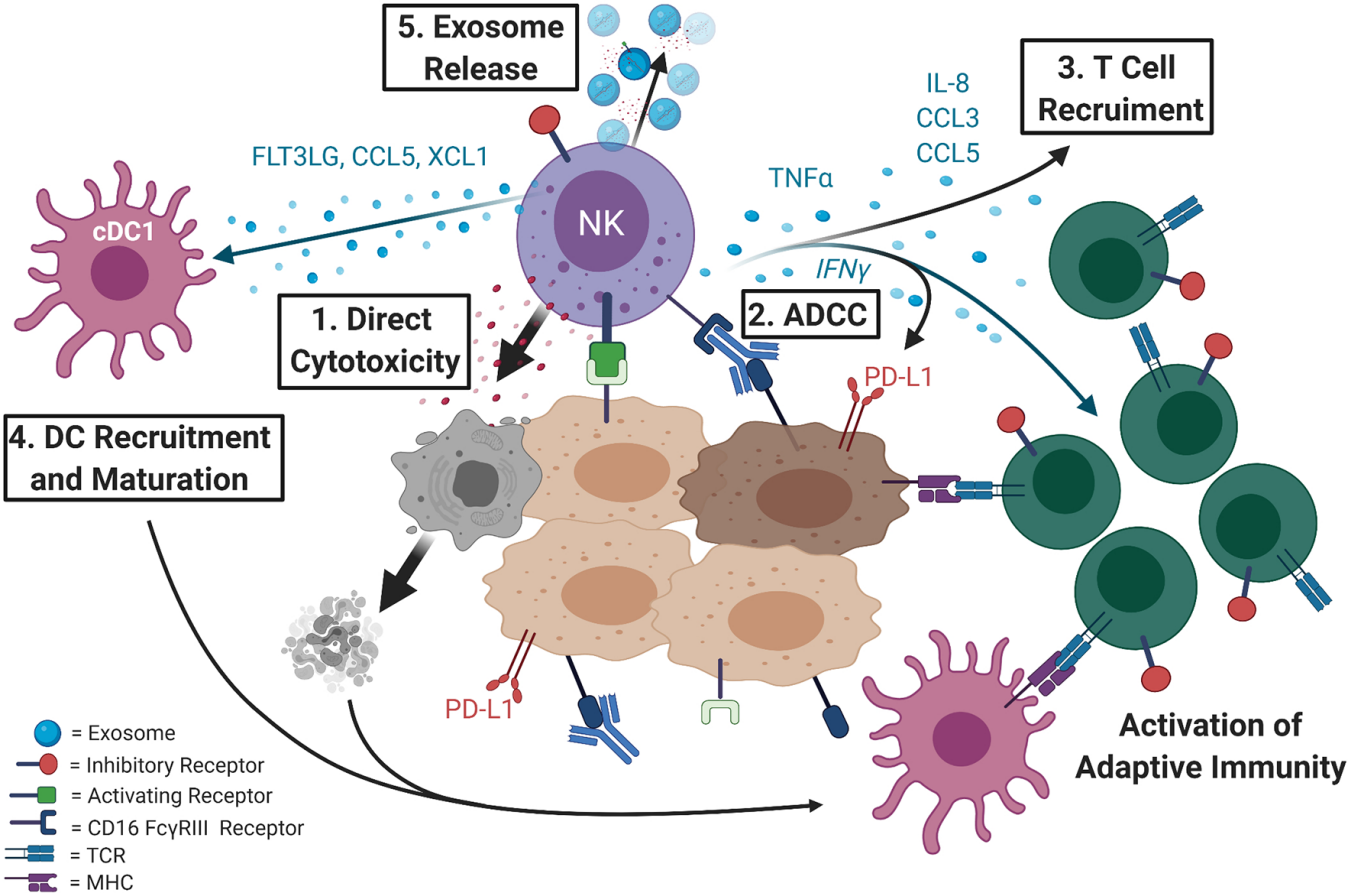
NK cells are key effectors of anti-tumor response and direct both the innate and the adaptive arms of the immune system. 1) NK cells are the first responders of the immune system and can directly recognize and lyse tumor cells. Activating receptors on NK cells recognize ligands that are mostly expressed on compromised cells while inhibitory receptors bind to self-ligands that mark healthy, normal cells. 2) NK cells also express the CD16 FcγRIII receptor that binds antibodies and triggers antibody-dependent cellular cytotoxicity (ADCC). This response contributes to efficacy of many of the antibody-based cancer therapeutics (e.g. Herceptin or Erbitux). 3) NK cells not only directly lyse compromised cells causing release of tumor antigens, but when activated release cytokines such as TNF-α and IFN*-γ*, the later known to induce PD-L1 expression, that can recruit other immune cells and inflame or “heat up” the tumor microenvironment priming it for immunotherapy. 4) Intratumoral NK cells produce chemoattractants CCL5 and XCL1 (5) as well as FLT3LG, the formative cytokine of rare intratumoral stimulatory dendritic cells (cDC1) (6) that can activate the adaptive immune response. NK cells have also been shown to directly recruit T cells by releasing cytokines such as IL-8, CCL3, and CCL5 (7). 5) Additionally, NK cells can release exosomes with cytotoxic activity and can contain effector miRNAs, cytokines, and display NK cell surface receptors.

Natural Killer (NK) cells are a small subpopulation of lymphocytes that are a part of the innate immune response and are key effectors of immunosurveillance and immunoregulation. NK cells are the first responders of the immune system and have an inherent ability to recognize and lyse virally-infected, stressed, or cancerous cells without prior sensitization or antigen presentation (**Figure 1**). NK cells perform this differential surveillance of malignant or compromised cells from normal “self” cells through the balance of signaling from surface activating receptors [*e.g.* NKG2D, natural cytotoxicity receptors (NCRs), 2B4, DNAM-1, activating killer cell immunoglobulin like receptors (KIRs)] and inhibitory receptors (e.g. inhibitory KIRs, NKG2A) that recognize a large repertoire of up- or downregulated molecules including major histocompatibility complex (MHC) class I chain-related proteins A and B molecules, and human leukocyte antigens (HLAs), nectin family proteins such as PVR and many others. The NK cell cytotoxic response is triggered when the activating signals are in excess of inhibitory signals (8). They also express the FcγRIII receptor (CD16) that recognizes antibodies to specific tumor antigens and triggers antibody-dependent cell-mediated cytotoxicity (ADCC) (**Figure 1**). Thus, rather than searching for one unique antigen on a target cell as the T cells do, NK cells recognize patterns of expression indicative of transformation into malignant cells. This broad recognition allows NK cells to preferentially kill tumor cells over healthy tissue without the need for prior training and without being dependent on one unique molecule that when downregulated could lead to a tumor escape from NK cell killing.

Cytotoxicity by NK cells is carried out by releasing cytoplasmic granules containing perforin and granzymes. However, NK cells not only directly kill compromised cells, but when properly activated, can be potent producers of TNF-α and IFN*-γ*, the last one being a known inducer of PD-L1 expression. Alternative mechanisms by which NK cells were shown to carry out their anti-tumor function involve expression of death receptor ligands FasL and/or TRAIL (9–12) and release of extracellular vesicles, such as exosomes, with cytotoxic activity (13) that contain effector miRNAs [reviewed in (14)], cytokines, and display NK cell surface receptors (15–17). In addition to direct killing, NK cells secrete chemokines and cytokines to recruit and coordinate responses by other immune cells, such as T cells (7) and dendritic cells (DCs), in the tumor microenvironment or site of infection and can prime the adaptive immune response for better viral or tumor control (5–7, 18–23) [reviewed in (24)] (**Figure 1**).

Recent studies have highlighted the importance of functional NK cells for the success of immunotherapies, including a critical role in successful PD-1/PD-L1 blockade treatment (25, 26). For example, presence of NK gene signatures defined by GNLY, KLRC3, KLRD1, KLRF1, NCR1 genes correlated with FLT3LG levels and presence of BDCA-3^+^ stimulatory DCs along with improved overall patient survival in all cancer types examined (6). Furthermore, in melanoma patients this study found NK cell frequency correlated with response to anti-PD-1 treatment and improved overall survival while no correlation was found for Treg cells, CD4^+^ T_H_ cells, CD8^+^ T cells and PD1^+^ CTLA4^+^ T cells (6). Similar correlation between higher density of intratumoral NK cells and response to therapy was found in a study of 25 patients with metastatic melanoma treated with anti-PD-1 (27). Studies examining the mechanisms of action of checkpoint inhibitors in humans and mice have shed light on the complex interface between the innate and adaptive immune responses, expanding the traditional NK cell functional domain. NK cells join DCs and not only bridge but rather orchestrate the innate and adaptive immunity. NK cells hold a distinct role in potentiating the innate immune response and activating the adaptive immune system through the secretion of pro-inflammatory cytokines and chemokines, including regulating T cell responses. NK cells can directly affect T cells by cell-to-cell contact, and indirectly by secretion of cytokines or by recruitment of DCs and modulation of antigen-presenting cells. NK cells can target activated T cells for elimination and promote differentiation of naïve CD4 T cells [reviewed in (28)].

Most cancer patients have NK cells that are dysfunctional or low in frequency and are further negatively impacted by surgery and standard chemotherapy treatments (29) [reviewed in (30)]. For example, dysfunction of NK cells can be caused by induction of the glycolysis-inhibiting enzyme fructose-bisphosphatase 1 (FBP1) which leads to tumor progression in KRAS-driven models of lung cancer (31). In this model at later stages, tumor growth could only be slowed by transfer of functional NK cells. Additionally, dysfunctional NK cell response can be caused by altered make up of proteins expressed on surface of tumor cells (31–34). For example, it was shown that radiation increased the expression of PD-L1 but decreased expression of activating ligands for NKG2D NK cell receptor through IL-6-MEK/ERK signaling in non-small cell lung cancer (NSCLC) cell lines, protecting the tumor cells from NK cell cytotoxicity (32). Thus, the lack of functional NK cells and/or effective NK cell response may be a potential cause behind the limited response to immunotherapies or other targeted therapies (e.g. therapeutic antibodies) that rely on NK cells for efficacy (31–34). To address this, adoptive NK cells therapies could provide a viable solution to increase response to immunotherapies (35, 36). Over the past decade advancements have been made to generate highly cytotoxic NK cells as an “off-the-shelf” cell therapy treatment that have the potential to mount a functional response in the setting of altered tumor environment that poses a critical barrier for endogenous NK cells. These cells can be further modified to enhance their targeting (e.g., with chimeric antigen receptors) and decrease their sensitivity to tumor immunosuppression (e.g. NKG2A knock-out). Thus, appropriate NK cell-based therapeutics could be effectively applied with immunotherapies to increase response rates and duration.

This review highlights the importance of the early actions of the NK cell response and the pivotal role NK cells hold in priming the immune system and setting the stage for successful response to cancer immunotherapy with focus on approved immunotherapies or those in late-stage clinical trials. The mechanisms that can drive NK cell dysfunction are reviewed with the intent to demonstrate how this can negatively impact subsequent immunotherapy response and how there is a need for prospective studies with focus on the role of NK cell compartment in immunotherapeutic response. The last part will highlight the advancements in NK cell therapeutics and how NK cell-based therapeutics can provide a viable solution to increase success of most immunotherapeutic therapies (and beyond).

## NK Cells and Immune Checkpoint Inhibition

The success of monoclonal antibodies targeted to block immune regulatory checkpoint receptors or ligands has shifted immune checkpoint inhibitors and immunotherapy to the forefront of oncology [reviewed in (37)]. A Phase III clinical trial (NCT01866319) of the checkpoint inhibitor Pembrolizumab (anti-PD-1) produced overall response rates (ORR) in 33% of patients with advanced-stage melanoma (38). Since then, Pembrolizumab has been indicated for the treatment of twenty cancer types (39) and a search of the NCT database using the keyword ‘Pembrolizumab’ showed over 100 Phase III or IV interventional clinical trials that are currently active determining the efficacy of Pembrolizumab in more cancer types and in combination therapies. Although there are reports of durable objective response rates for many patients, the overall response rates are still low, and many patients eventually relapse. For example, in the ongoing Phase IB clinical trial NCT02054806 studying the efficacy of pembrolizumab in patients with advanced solid tumors, while treatment of patients with some tumor types have resulted in preliminary overall response rates over 30%, most are much lower (40–42). Preliminary results from NCT02054806 and the completed Phase 1 Clinical Trial NCT01848834, showed Pembrolizumab treatment of patients with colorectal cancer resulted in ORR of only 4.3% (43), while for patients with triple negative breast cancer, head and neck squamous cell cancer, gastric cancer, and urothelial carcinoma the ORR were between 15.6% and 21.2% (40, 44–47). Thus, the scope of the clinical success of immune checkpoint blockade therapies is limited to a select subset of patients typically with cancers expressing high levels of PD-L1 and infiltrated with lymphocytes. Current strategies have focused on combination therapies with PD-1 checkpoint inhibitors, and in fact over 3000 clinical trials are ongoing (48). Many of these combination therapies are showing success. For example, the PD-L1 inhibitor Atezolizumab plus Tiragolumab, an anti-TIGIT antibody (TIGIT is a highly expressed receptor both on T cells and NK cells) has shown early clinical activity with on ORR of 46% in patient with advanced solid tumors and is currently in phase I clinical trials (49). Many checkpoint inhibitor combinations have failed as well. Understanding mechanistically the variables contributing to the heterogeneity of response to checkpoint blockade is necessary for better rational design of these therapies in order to increase efficacy of combination therapies and to achieve more widespread responses and/or longer response duration.

Current strategies to improve immune checkpoint blockade therapies predominantly focus on cytotoxic CD8^+^ T cells, however emerging evidence suggests contributions from other immune cells to the efficacy of checkpoint inhibitors. Many cancer types have adopted mechanisms to suppress and evade detection by the immune system, commonly through the loss of major histocompatibility complex (MHC) molecules or depressed neoantigen load [reviewed in (50)]. While down regulation of MHC expression may render tumors camouflaged from detection and lysis by CD8^+^ T cells, tumors that express high levels of PD-L1, even with lower MHC expression, are still responsive to PD-1/PD-L1 blockade (51, 52). These findings challenge the prevailing view that T cells are the exclusive mediators of the anti-tumor response and suggest the involvement of other immune cell populations that are also unleashed by PD-1 blockade and provide a critical support for the overall success of the treatment. Recent studies have supported this idea that multiple effector cell populations, including NK cells, are impacted by immune checkpoint inhibition and treatment efficacy hinges on the collective contributions of these populations (53, 54) [reviewed in (55) and (56)].

NK cells share similar effector functions and roles as cytotoxic T cells but are able to direct the immune response towards resistant tumor cell populations. Contrary to CD8^+^ T cells, loss of MHC removes inhibitory interaction with KIRs on NK cells and thus makes tumor cells more susceptible to lysis by NK cells. Thus, NK cells have shown to be uniquely capable of targeting highly aggressive cancer stem-like cells and undifferentiated tumors, which are highly refractory to chemotherapy. In addition, NK cells are capable of catalyzing differentiation of tumor cells *via* secreted and membrane-bound IFN*-γ* (57). Differentiation prompts remodeling of the surface receptor profile – an increase in MHC class I and CD54 and decrease in CD44 expression – and reins in tumor growth and metastasis (58). These differentiated tumors should be also better targets for T cell recognition and elimination. NK cells have been shown to selectively target senescent tumor cells. A study led by Ruscetti et al. determined that the observed reduced proliferative capacity of KRAS-mutant lung tumors in mice treated with a cytostatic drug regimen resulted primarily from the natural senolytic activities of NK cells (59). Although NK cells have been less heavily studied in the context of checkpoint blockade, current evidence supports NK cells involvement and impact on the response to immunotherapy.

### NK Cells in PD-1/PD-L1 Checkpoint Blockade

**Figure 2:**
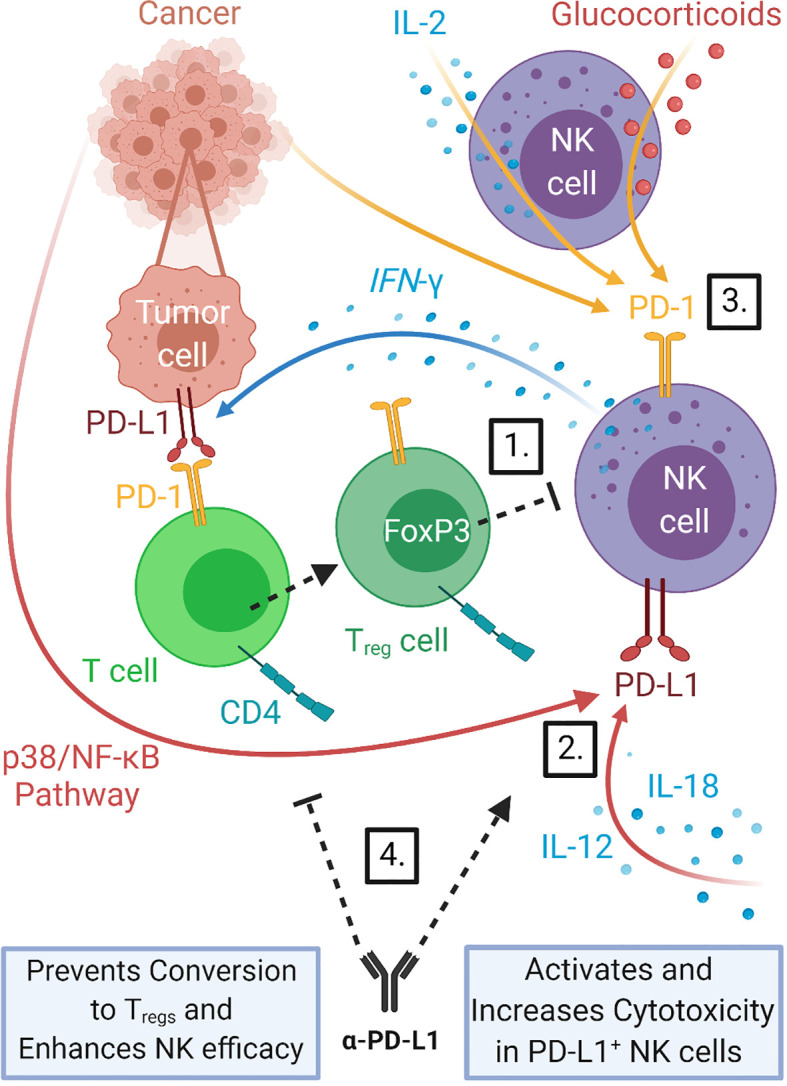
NK cells interact with the PD-1/PD-L1 immune checkpoint axis. NK cells can increase the expression of PD-L1 on tumor cells through release of cytokines such as IFN*-γ*, promoting PD-1/PD-L1 driven stimulation of Treg production which in turn can inhibit NK cell function. 1) NK cells have also been shown to express both PD-L1 and PD-1 themselves. PD-L1 expression can be induced in NK cells by direct interaction with tumor cells *via* the p38/NF-κB pathway and by stimulation with cytokines IL-12 and IL-18 (25). 2) PD-1 expression in NK cells has been shown to be upregulated in a variety of cancers (26, 60, 61) and to be inducible in response to IL-2 stimulation (60) and glucocorticoid signaling (62). 3) Treatment with PD-1/PD-L1 blockade therapy can help prevent Treg inhibition of NK cells and counteract PD-1/PD-L1 driven NK cell dysfunction. 4) PD-L1 expression on tumors correlates with response to PD-1/PD-L1 checkpoint blockade therapies, thus induction of PD-L1 by NK cells should improve outcomes of this treatment.

The presence of PD-L1 in tumors has been shown to be a predictor of tumor response to PD-1/PD-L1 checkpoint blockade and NK cells have an intricate interplay with the PD-1/PD-L1 axis. NK cells have been shown to increase PD-L1 expression on tumor cells, express PD-L1 and PD-1 in some contexts and be directly inhibited by interaction with PD-L1 positive tumors or indirectly by changes in the tumor milieu in response to PD-L1 induction. Additionally, blockade of the PD-1/PD-L1 axis have been shown to increase NK cells anti-tumor response. This is summarized in **Figure 2** and discussed in detail below.

Melanoma patients who responded to anti-PD-1 therapy had higher intratumoral and peritumoral NK cell densities, and these NK cells had increased cytotoxic signatures of elevated CD16 expression and granzyme B versus NK cells in non-responders (27). Activated NK cells are a major source of IFN*-γ*, which drives cancer-induced inflammation and leads to induction of PD-L1 expression on tumor cells. As an example, particle activated NK cells (PM21-NK cells) were shown to induce PD-L1 on tumors both *in vitro* and *in vivo* (63). Presence of PD-L1 on tumors has been so far the most reliable marker of treatment response (64) and is used for patient selection for treatment of NSCLC. Presence of PD-L1 expression on tumors is typically associated with improved response to anti-PD-1/PD-L1 treatment (65, 66). In fact, Avelumab failed to show survival advantage over docetaxel in patients with platinum treated NSCLC when all patients (i.e., with PD-L1 tumor expression of ≥1%) were included but survival advantage was observed in exploratory analysis when patients were stratified based on PD-L1 expression on their tumors (65). Median survival was 10.5 months (95% CI 9.2-12.9) in the entire Avelumab group with the PD-L1 ≥1% *versus* 9.9 months (8.1-11.8) in the docetaxel group, but in stratified analysis of the Avelumab group median survival was 13.6 (10.1-18.5) when PD-L1 expression cutoff was set to ≥50% and 17.1 (10.6-25.0) with cutoff of ≥80% (67). NK cells as the first responders are likely one of the main populations that drives the induction of PD-L1 on tumors yet, as will be discussed in detail in later section, are frequently dysfunctional in cancer patients. Adoptive transfer of activated NK cells with high IFN*-γ* could potentially improve response to PD-1/PD-L1 blockade *via* induction of PD-L1.

As described above PD-L1^+^ tumors show favorable responses to PD-L1 blockade, however responses were also observed for patients with tumors lacking PD-L1 expression (68, 69). PD-L1 can be expressed on cells other than tumors including on immune cells such as e.g. dendritic cells or myeloid derived suppressor cells within the tumor microenvironment and thus inhibiting anti-tumor response by effector immune cells (70). Anti-PD-1/PD-L1 treatment can lead to reactivation of inhibited effector cells with subsequent IFN-γ secretion as a result of an anti-tumor response (71) and likely induction of PD-L1 on initially PD-L1^-^ tumor cells. Recent publication by Dong et al. identified PD-L1^+^ NK cells as the cytolytic effector cell population that may provide alternative explanation to the efficacy of anti-PD-L1 antibody therapy in these settings where tumors lack PD-L1. PD-L1 expression is inducible on activated NK cells through direct interaction with tumor cells *via* the p38/NF-κB pathway and by stimulation with cytokines IL-12 and IL-18 (25). *In vitro*, PD-L1^+^ NK cells display heightened cytotoxicity compared to their PD-L1^-^ counterparts which is further enhanced by engagement with anti-PD-L1 antibodies. In response to anti-PD-L1 treatment with Atezolizumab, mice engrafted with human NK cells and PD-L1^-^ K562 myeloid leukemia cells demonstrated significantly elevated levels of granzyme B, *IFN-γ*, and CD107a, contributing to notable reductions in tumor burden and significant improvement in survival over the placebo controls (25). Survival advantages were lost both in mice lacking PD-L1^+^ NK cells and in NK cell-depleted mice. Congruent with the above findings, acute myeloid leukemia (AML) patients who achieved complete remission were found to have a higher proportion of PD-L1^+^ NK cells at complete remission compared to at the time of diagnosis as well as compared to AML patients who failed to reach complete remission (25). Taken collectively, these studies suggest the PD-L1 status of NK cells should be an important consideration in determining the efficacy of PD-1/PD-L1 checkpoint blockade therapy.

Effector cells such as T cells have been shown to benefit from checkpoint blockade through the inhibition of the PD-L1 receptor PD-1 on their surface. NK cells isolated from healthy donors do not constitutively express PD-1, however PD-1 expression is inducible in response to IL-2 stimulation (60) and glucocorticoid signaling in the stress response has been linked to PD-1 upregulation on NK cells (62). PD-1 has also been found to be upregulated on activated NK cells in a variety of cancers with mean expression levels ranging widely from 9% to 64% dependent on the cancer setting (26, 60, 61). Yet, PD-1 expression on NK cells appears to be context dependent and thus different observations were made dependent on the experimental conditions used. A recent study that extensively examined the PD-1 expression on NK cells from human and mouse in context of tumor and viral models found that as opposed to T cells, NK cells mostly lacked PD-1 expression arguing for a more indirect interaction with the PD-1/PD-L1 axis (72). More in-depth studies on the mechanisms regulating PD-1 receptor expression on NK cells are needed.

The presence of PD-1 on NK cells affects their function. While PD-1 expression on NK cells can initially activate them, it drives expression of PD-L1 on tumor cells which can lead to NK cell exhaustion. PD-1^+^ murine NK cells compared to PD-1^-^ murine NK cells demonstrate an activated signature, characterized by expression of NK cell activation markers SCA-1 and CD69, CD107a expression, and intracellular accumulation of IFN*-γ* after tumor engagement (26). However, PD-L1 ligation leads to dysfunction of IL-2-activated PD-1^+^ NK cells, marked by blanket downregulation of CD16 and CD107a (61), transitioning NK cells from an activated to exhausted phenotype. To combat this, immunomodulation *via* the PD-1/PD-L1 axis checkpoint inhibitor Nivolumab was shown to restore cytotoxicity of PD-1^+^ NK cells co-cultured with tumors expressing high levels of PD-L1 (27, 61) and reestablish the IFN*-γ* response of NK cells (61). Restoration of NK cell faculties correspond with improved clinical outcomes in head and neck cancer patients (61).

As suggested earlier, even in context where NK cells lack PD-1 or PD-L1 expression, checkpoint inhibitors can also indirectly influence anti-tumor NK cell functions through the modulation of other immune cell populations (63, 73) (**Figure 2**). Crosstalk between CD4^+^ T cells and NK cells is requisite for optimal NK cell activity. CD4^+^ T cells activate NK cell function two-fold: directly through the secretion of stimulatory IL-2, and indirectly by stimulating antigen presenting cells to secrete IL-12, with both cytokines working synergistically to positively regulate IFN*-γ* production by NK cells. The importance of CD4^+^ T cell/NK cell interaction is highlighted by the observed correlation between CD4^+^ T cell exhaustion in chronic infections and impaired NK cell-mediated lysis of target cells (74). PD-1 signaling on T cells is independently capable of converting CD4^+^ T helper cells into regulatory T cells (Tregs) by inducing Foxp3, a transcription factor that drives this conversion and is critical in the maintenance of immunosuppressive Treg functions (75, 76). Surface expression of transforming growth factor beta 1 (TGF-β) on CD4^+^ CD25^+^ Foxp3^+^ Tregs controls the expression of key NK cell activation receptors – NKp30, NKG2D, and CD16 (77–80)– and neutralizes the potent anti-tumor NK cell response. There is a dual effect amplifying the negative impact of PD-L1 on NK cells whereby expanded Tregs directly inhibit NK cells and also their expansion depletes CD4^+^ T cells and thus diminishes the positive effects of CD4^+^ T helper cells on NK cell function. PD-1/PD-L1 blockade indirectly offers improved NK cell survival and function by preventing the expansion and persistence of inhibitory Tregs in the tumor microenvironment (63) and potentially by mitigating CD4^+^ T helper cell exhaustion. Accordingly, PD-L1 blockade enhanced anti-tumor efficacy of expanded PD-1^-^ NK cells that were previously otherwise unaffected by anti-PD-L1 treatment *in vitro*. CD4^+^ T cell exhaustion is mediated dually by IL-10 and the PD-1. In the context of an HIV infection, combined blockade of the PD-1 and IL-10 pathways reinvigorates CD4^+^ T cell effector functions, resuming NK cell degranulation and cytolysis (74). This strategy of immunomodulation boosting CD4^+^/NK cell cooperativity may prove beneficial in cancer therapy. Sequestration of IL-2 by Tregs *via* their high-affinity IL-2R receptor is an alternative mechanism by which Tregs weaken NK cell anti-tumor activities (81). Various strategies to engineer recombinant human IL-2 that is biased toward low-affinity IL-2 receptors present on NK and CD8^+^ T cells in efforts to mitigate Treg-driven immunosuppression have been developed (82–84). They are currently being tested in Phase I and II clinical trials after yielding promising results in murine models, both alone and as combination partners for checkpoint inhibitors in cancer therapy (82–84).

Rejuvenation of impaired NK cell activity holds broader implications regarding the immune response as NK cells are involved in the priming of the adaptive immune system *via* recruitment of other immune cells, such as DCs. NK-DC cross-talk is an important interaction involved the innate immune response. Cross-talk between NK and DC cells leads to DC maturation and NK cell activation. NK cells release IFN-γ and TNFα which promote DC maturation (85). In turn mature DCs can secrete cytokines such as IL-12 and IL-15 that stimulate NK cell proliferation and survival and IFN-γ production (85, 86). Activated NK cells also have the ability to kill DCs that do not properly mature by engagement of the activating receptor NKp30, term DC editing (87). DC-NK cross-talk is an important player in the immune response to tumors and should be considered in evaluating the effects of cancer immunotherapy. This topic has been widely investigated in recent years, see (24, 88) for a review of this topic. Conventional type 1 dendritic cells (cDC1) serve in cross-priming T cells in tumor-draining lymph nodes through the secretion of chemo- and cytokines regulating T cell survival, effector functions, and their trafficking to the tumor microenvironment. The importance of cDC1 in oncologic immunity is highlighted by the abolishment of tumor rejection and responsiveness to adoptive T cell therapy and immune checkpoint blockade in mice lacking cDC1 (89, 90) and it has been shown that induction and activation of tumor-residing cDC1s can help overcome resistance to anti-PD-L1 therapy (91). Activated NK cells are paramount in producing cDC1 chemoattractants and mobilizing them to the tumor microenvironment, which in turn recruit T effector cells and launch the adaptive immune response (5) (**Figure 1**). Increased presence of both NK and intratumoral cDC1 cell populations, and not T cells, in the tumor microenvironment was a predictive biomarker of tumor responsiveness to anti-PD-1 immunotherapy and prolonged overall survival in melanoma patients (6). In support of this, new evidence challenges the widely accepted theory that PD-1 blockade reinvigorates pre-existing, exhausted, tumor-infiltrating T cells and suggests that *de novo* recruitment of T cells is the main mechanism of PD-1 blockade. Comparing single-cell RNA sequencing and T cell receptor (TCR) sequencing data of tumor-infiltrating T cells before and after PD-1 blockade in patients with basal cell or squamous cell carcinoma, Yost et al. found that tumor-infiltrating TCR clones present prior to administration of PD-1 blockade are neither activated nor enriched in the tumor microenvironment following treatment (92). Rather, the prevailing T cell population present post-treatment expresses novel TCR specificities not identified in the pre-treatment tumor sample, suggesting anti-PD-1 therapy does not reactivate existing exhausted tumor-infiltrating T cells, but rather recruits new, activated T cells from the peripheral blood to the tumor (92). Given the seminal role NK cells hold in directing the adaptive immune response outlined above, it is likely NK cells are responsible for the recruitment of novel T cells to the tumor microenvironment. Verification of this hypothesis in future studies would add to the evidence that NK cells are important early organizers of the body’s anti-tumor response.

Collectively, these findings provide evidence that PD-1 is an important checkpoint in NK cell activation acting upon NK cells *via* multiple direct and indirect mechanisms summarized in **Figure 2** and that PD-1/PD-L1 immunotherapy not only revives NK cell-mediated lysis of tumor cells and cytokine production, but concurrently supports the NK cell-directed priming and recruitment of the adaptive immune response. Thus, addition of adoptive NK cell therapy to treatments targeting PD-1/PD-L1 axis has the potential to improve outcomes. In support of this, results from a completed Phase II clinical trial of combination PD-1 inhibitor Pembrolizumab and allogeneic *ex vivo* expanded NK cells showed significant improvement of survival of patients with previously treated advanced NSCLCs that received combination therapy as compared to Pembrolizumab alone (93). Phase I/IIa clinical trial (NCT03937895) for combination therapy of allogenic NK cells and Pembrolizumab is ongoing for treatment of biliary tract cancer.

### NK Cells in CTLA-4 Checkpoint Blockade

Another breakthrough checkpoint therapy relies on targeting the CTLA-4 molecule, also known as CD152. Ipilimumab is a highly successful antibody against CTLA-4 approved by the FDA for treatment of melanoma [reviewed in (94)] and for combination therapy with Nivolumab (anti-PD-1) for advanced renal cell carcinoma, MSI-H/dMMR metastatic colorectal cancer, hepatocellular carcinoma, metastatic NSCLC, and malignant pleural mesothelioma (95) [reviewed in (96)]. CTLA-4 is an inhibitory receptor constitutively expressed in Tregs and upregulated in activated T cells. Stojanovic et al. found that CTLA-4 and the T cell activating receptor CD28 also regulate the NK cell response in mice (97). CD28 and CTLA-4 are found to be upregulated in murine NK cells in response to IL-2 activation(97). These receptors work antagonistically to regulate IFN-γ production by NK cells: CD28 promoting IFN-γ synthesis while CTLA-4 suppresses it.

A correlation between higher frequencies of CTLA-4^+^ Tregs in the tumor microenvironment and abrogated NK cell activation and cytotoxicity in head and neck cancer patients treated with Cetuximab (anti-EGFR) was reported by Jie et al. (98). Anti-CTLA-4 pathway blockade mediates selective depletion of CTLA-4^+^ tumor-infiltrating Tregs and could therefore indirectly rescue NK cells from Treg suppression. This evinces that NK cells are also potential targets of CTLA-4 blockade (99)*. A*nti-CTLA-4 was effective in eliminating intratumoral Tregs and initiating the recovery of NK cell ADCC following Treg suppression (98, 100). Combinatorial administration of anti-CTLA-4 with IL-2Cx, a complex of IL-2/anti-IL-2 which directs IL-2 to NK and CD8^+^ T cells but not Tregs, or IL-15/IL-15Ralpha complexes further tips the tumoral effector/regulatory cell ratio in favor of activated NK cells and enhances tumor control (82, 101). A Phase I clinical trial (NCT04290546) is ongoing to evaluate combination therapy of Ipilimumab, IL-15 superagonist N-803, and adoptive NK infusion for head and neck cancer.

### NK Cells in NKG2A Blockade

As opposed to PD-1 and CTLA-4, the inhibitory NKG2A receptor is expressed predominately on NK cells and a select subset of CD8^+^ T cells has also been identified as a prospective target for this checkpoint blockade. Engagement of human leukocyte antigen-E (HLA-E) by the NKG2A receptor sends a strong signal inhibiting NK cell-mediated lysis of the target cell (102). Upregulation of the NKG2A ligand, HLA-E, by malignant cells in response to IFN-γ secreted by tumor-reactive immune cells is a common mechanism by which tumors thwart NK cell surveillance (103). NKG2A signaling blockade or downregulation of NKG2A receptor expression should bypass HLA-E-induced NK cell inhibition and restore normal NK cell function. In vitro studies showed anti-NKG2A Monalizumab treatment prompts increased CD107a expression, a marker for activated NK cells, and IFN-γ production by IL-2 activated NK cells and CD8^+^ T cells, yielding significant improvements in tumor growth control and prognosis (104). The beneficial effects observed with anti-NKG2A blockade are magnified when used in conjunction with anti-PD-1 Durvalumab (104). Furthermore, combining anti-NKG2A Monalizumab with anti-EGFR Cetuximab was shown to promote ADCC, evidenced by the higher density of CD137 activation markers on NK cells (104).

Two recent studies that used engineered NK cells lacking functional NKG2A underscore that NKG2A is a critical inhibitor of NK cell responses, and an important target for immunotherapies. A new study by Berrien-Elliott et al. has shown NKG2A is transcriptionally induced in cytokine-induced memory-like NK (CIML NK) cellular therapy and a dominant checkpoint, but not in conventional NK cell anti-tumor response (105). Anti-NKG2A treatment or NKG2A knock-out returned CIML NK IFN-y production and response to HLA-E+ K562 cells. NKG2A blockade or elimination also restored CIML NK cell anti-leukemia response. Secondly, Kamiya et al. (106). engineered NK cells to express single-chain variable fragment from an anti-NKG2A antibody linked to an endoplasmic reticulum-retention domain (106). This approach prevents nascent NKG2A from migrating out of the endoplasmic reticulum, effectively blocking its *de novo* expression. Experiments using immunodeficient mice engrafted with Ewing’s sarcoma or osteosarcoma cell lines transduced with HLA-E found that the majority of immunodeficient mice receiving NKG2Anull NK cell infusions achieved long-term survival, with the median overall survival exceeding 269 days following Ewing’s sarcoma injection and median survival not reached after 60 days follow-up for osteosarcoma injection (106). Control NK cells only delayed tumor development with median survivals of less than 40 days (106). These two studies highlight the importance of NKG2A blockade and present this NK inhibitory receptor as an important target for future immunotherapeutics. In fact, the safety and efficacy of adjunct therapy combining Monalizumab with Cetuximab is currently being assessed in a Phase II clinical trial (NCT02643550) in patients with recurrent or metastatic squamous cell carcinoma of the head and neck.

### Other Checkpoints

**Figure 3:**
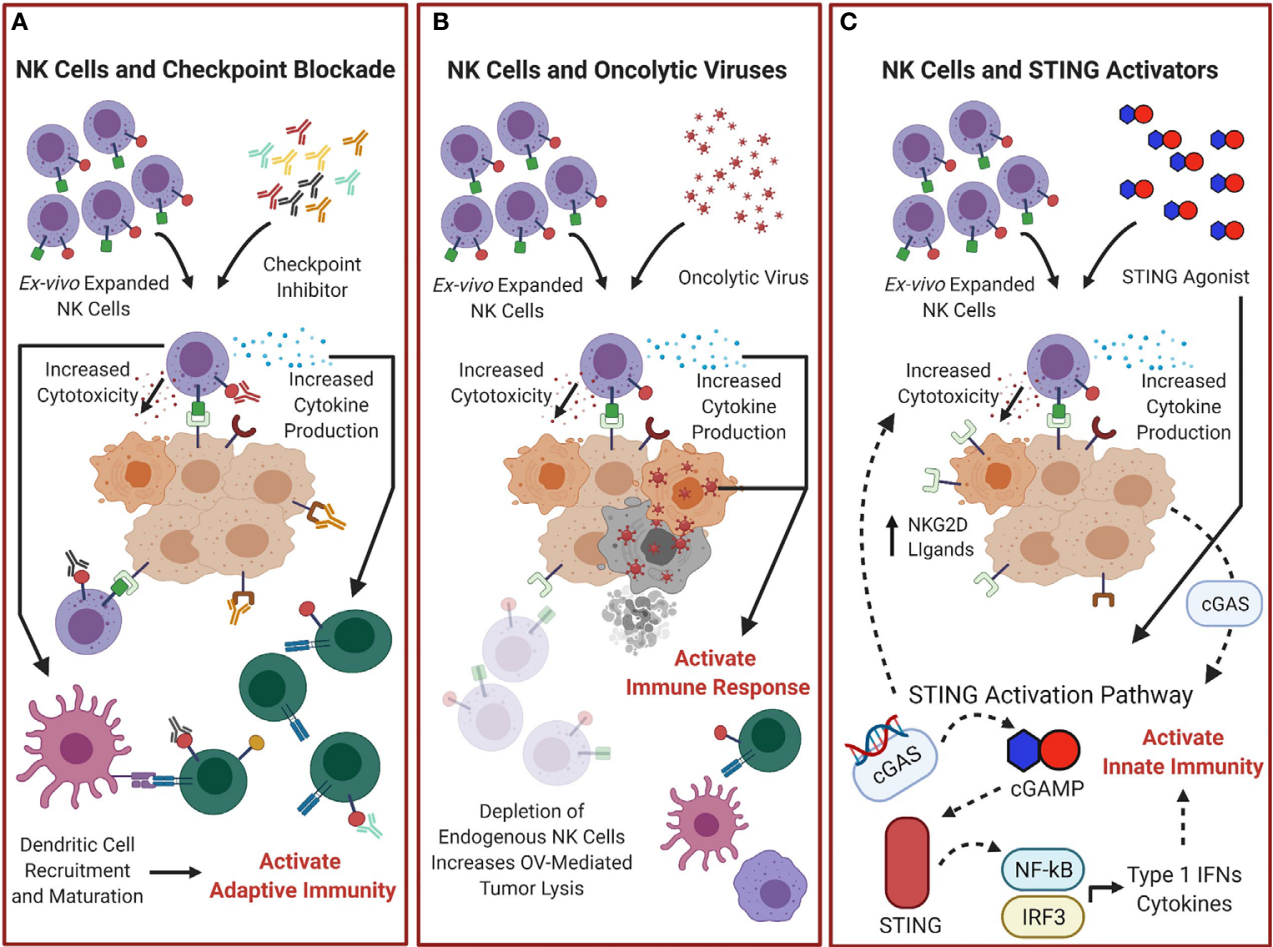
Combination treatments of adoptive NK cells with other Immunotherapies could improve outcomes. **(A)** Adoptive transfer of NK cells combined with checkpoint inhibitor blockade could increase overall NK cytotoxicity and cytokine production and help control tumor and activate the adaptive immune response. **(B)** NK cell therapy combined with oncolytic virotherapy (OV) could improve therapeutic efficacy. Depletion of endogenous NK cells would reduce the natural antiviral response and increase OV mediated tumor lysis, and adoptive transfer of NK cells would increase NK cell effector functions and enhance the antitumor response. **(C)** STING-dependent tumor rejection activated by cGAS expression from tumor cells (107) can be enhanced by combination therapy of STING agonists with NK cells. This would provide enhanced tumor lysis through further activation of the STING pathway, not only activating the innate immunity by stimulating expression of cytokines and Type I IFNs, but by increasing the presence of NK cell activating receptors ligands, which could enhance adoptive NK cell therapy antitumor responses.

Many therapeutics targeting other immune checkpoint are being developed and evaluated clinically and are reviewed elsewhere. Some of these checkpoints are highly expressed on NK cells such as IL-1R8, TIGIT, TIM-3, and KIRs and the efficacy of therapies targeting these molecules will depend on the functional state of NK cells. Future studies to evaluate combination therapy of these inhibitors with adoptive NK cell transfer could provide methods for enhanced cancer treatment and tumor control. The role adoptive NK therapy plays in the use of checkpoint inhibitors is summarized in **Figure 3A**.

## NK Cells and Oncolytic Viruses

Oncolytic viruses (OVs) are a novel class of drugs that are rapidly gaining traction in cancer treatment. Exploiting cancer cells’ defective antiviral defenses, viral replication within cancer cells causes cell lysis. In 2015, the field marked a major milestone with the first FDA-approved oncolytic virus, Talimogene laherparepvec, a modified type I herpes simplex virus, for treatment of advanced melanoma [reviewed in (108)]. Numerous other clinical trials are currently ongoing. Initially therapeutic efficacy of OVs was thought to be derived solely from the direct killing of tumor cells. Now, the field recognizes a split mechanism of OV action: in addition to direct lysis of cancer cells, induction of the adaptive and innate immune system by OVs largely contributes to the observed efficacy of OV agents, questioning the previously held belief that pre-existing antiviral immunity poses a major impediment to this treatment modality. Moving forward, a better understanding of the interplay between the established immune system and OVs is necessary to optimize antitumor immunity and improve therapeutic interventions.

In addition to their role in oncolysis, OVs prime the immune system to overcome the suppressive pressures of the tumor microenvironment. Fujihara et al. show that intratumoral injection of an inactivated Sendai virus (hemagglutinating virus of Japan-Envelope; HVJ-E) in mice enhanced local production of the IFN-inducible chemokine CXCL10 by DCs, which promoted intratumoral trafficking of activated IFN*-γ*-secreting NK cells and led to a reduction in renal cell carcinoma growth (109). In a follow up mouse study, systemic administration of IL-12-conjugated HVJ-E was found to further appreciate regional IFN*-γ* production and the magnitude of cytotoxic T lymphocyte activation (110). The resultant recruitment of activated innate and adaptive lymphocytes into the tumor milieu due to OV-mediated inflammation transitioned immunologically “cold” tumors into “hot” tumors that are responsive to immunotherapy. Conditioning of the tumor microenvironment and immune system reveals a significant corollary of therapeutic delivery of OVs and provides the rationale behind adjuvant oncolytic virotherapy.

NK cells are potentially a clinically relevant determinant of the therapeutic efficacy of oncolytic virotherapy. In a recent report by Leung et al. NK cells show contact-dependent activation and anti-cancer cytotoxicity against adenovirus-infected ovarian cancer cells (111). The immune system activation cascade is set into motion by the antiviral response of the NK cell compartment. In a study by Ricca et al. when testing if pre-existing immunity to Newcastle Disease Virus (NDV) increases the therapeutic efficacy of the oncolytic virus, they found that depletion of NK cells prior to initial immunization to NDV, decreased the therapeutic efficacy of NDV against tumors and NK cells are likely important for early tumor clearance, and recruitment and activation of CD8 T cells (112). However, given NK cells’ dual role in the body’s innate defense against malignancies and virally compromised cells, killing of virus-infected cancer cells by NK cells could also limit the extent of viral oncolysis and thus tumor clearance. A mathematical model developed by Kim et al. sheds light on how exogenous NK cell therapy would affect the use of OVs. Using a combination therapy coupling oncolytic herpes simplex virus and bortezomib, a proteasome inhibitor that amplifies viral replication, the model predicts both depletion of endogenous NK cells and injection of exogenous NK cells would yield enhanced antitumor efficacy (113). Depletion of endogenous NK cells reduces the friction applied by the antiviral immune response, increasing OV-mediated lysis of tumor cells, whereas, adjuvant injection of exogenous NK cells grants an advantage to the immune system, boosting tumor cell killing by NK cells (114). These predictions were validated in primary glioma mouse models, granting a significant survival advantage to mice receiving either endogenous NK cell depletion or exogenous NK cell injection. The combination of oncolytic measles vaccine virotherapeutics with activated human NK cells led to enhanced sarcoma cell lysis and increased NK activation markers (115) and provides further justification for clinical trials to test this combination therapy. Engineering of NK cells and OVs have also been suggested to further enhance the combinatorial therapeutic potential. Blockade of NK inhibitory receptor TIGIT was shown to increase the activity of adenovirus in ovarian cancer (111). Enhanced efficacy was seen when matching chemokine and receptor were incorporated into NK cells and vaccinia virus (116). CCR5-engineered NK cells combined with CCL5-expressing oncolytic vaccinia virus enhanced NK cell homing and therapeutic effects (116).

Combinations of OVs with other immunotherapies could have enhanced therapeutic benefits. Initial studies evaluating combination OV and checkpoint blockade therapy in mice generated data that underscores the notion that the therapeutic efficacy of oncolytic virotherapy is primarily contrived from the tumor-specific immune response coordinated by NK cells and carried out by CD8^+^ T cells, rather than direct virus-mediated lysis. Several pre-clinical studies describe potent therapeutic synergy when OVs and checkpoint inhibitors were administered jointly in mice (112, 117). The use of combinatorial OV and immune checkpoint therapy as well as engineering OVs for delivery of immune checkpoint inhibitors into the intratumoral environment is currently being investigated (118–122). Testing has also progressed to early-phase clinical trials and early reports remain promising (123, 124). Future research directed at probing the therapeutic variables including the nature of the virus, the checkpoint inhibitor, cancer setting, and dosing regimen and the impact adoptive NK cell therapy could have on these variables, and the identification of response biomarkers are necessary to optimize this multimodal therapy. The interaction between adoptive NK cell therapy and OV treatments is summarized in **Figure 3B**.

## NK Cells and STING Activators

Stimulation of interferon genes (STING) is a relatively new immunotherapeutic strategy. STING is a transmembrane protein localized to the endoplasmic reticulum that was first discovered as a cytosolic DNA sensor. Sources of cytosolic DNA can be nuclear, mitochondrial, or exogenous in origin. Tumors have a high incidence of chromosomal instability, driving the formation of micronuclei. These micronuclei can rupture and release DNA into the cytosol. Binding of cytosolic DNA and cyclic guanosine monophosphate (GMP)-adenosine monophosphate (AMP) synthase (cGAS) generates the STING-activating second messenger cyclic GMP-AMP (cGAMP) (125). STING activation produces NF-κB and interferon regulatory factor 3 (IRF3) which induce the transcription of type I IFNs (IFN*-α* and IFN*-β*) and other chemokines and cytokines that activate innate immunity (126, 127). Recently, STING activation has also demonstrated its essential involvement in priming NK cell-mediated antitumor immune responses. STING is an absolute requirement for the rejection of tumor cells that are sensitive to NK cell lysis and NK cell depletion abolished any STING-mediated protection in mice with RMA-S lymphoma or B16-BL6 melanoma (107). Intriguingly, Marcus et al. also found that cGAS expression by tumor cells, and therefore, tumor-originating cGAMP, is compulsory for STING-dependent tumor rejection (107). Strong relationships were observed between cGAS expression and NKG2D ligands (107). These findings are consistent with data previously reported by Lam and colleagues that activation of the cGAS-STING pathway increases expression of RAE-1 ligands for the activating NKG2D receptor on NK cells (128). Inactivation of cGAS in some tumors may serve as a mechanism of STING-mediated immune escape (129, 130) and delivery of exogenous cGAMP or STING agonists may stimulate intrinsic STING signaling, disabling cGAS-deficient tumor-driven immune suppression [reviewed in (131)].

The clinical significance of the cGAS-STING pathway was investigated in a gastric cancer by Song et al. The group observed a positive correlation between low STING expression and several clinical factors including tumor size, TNM stage, and patient survival (132). The group’s findings parallel conclusions from Marcus et al. that elevated cGAS expression positively correlated with prolonged survival in melanoma patients (107, 126, 127). These reports indicate that STING expression may be a useful prognostic tool, further evaluated in multiple tumor types in a recent study by An et al. (133). Moreover, STING agonists may “heat up” tumors, functioning as a precursor to immune checkpoint inhibitor treatment. STING activation catalyzes type I IFN production, stimulating the release of CXCL9 and CXCL10, which, as previously mentioned, draft the prerequisite tumor-infiltrating lymphocytes and precondition the immunological landscape for a robust checkpoint inhibitor-led anticancer response. In pre-clinical studies, intratumoral administration of the STING agonist, ADU-S100 (S100), provoked potent antitumor responses (134–136). Treatment of mice bearing poorly immunogenic B16 tumors with co-administration of S100, anti-PD-1, and anti-CTLA-4 yielded significant increases in IFN*-γ*-secreting tumor-specific T cells and conferred a significant survival benefit over mice receiving single-agent regimens (136). Despite CXCL9 and CXCL10 recruiting both NK cells and T cells, these studies predominately examined the antitumor impact of combined STING agonists and immune checkpoint blockade from a T cell perspective. The contributions of NK cells are often overlooked, however, based on our knowledge of the overlapping immunological niches of NK cells and T cells, it is not unreasonable to hypothesize that NK cell behavior mirrors that of T cells in these settings. A recent publication highlights the role of NK cells in response to STING agonists, showing STING-activating cyclic dinucleotides induce NK cell mediated tumor rejection in several tumor models independent of CD8^+^ T cells (137). Overall, further investigation aiming to uncover additional contributions the STING pathway may add in optimizing immunotherapy treatments and the effects on NK cell therapy are needed. **Figure 3C** summarizes these potential effects.

## Mechanisms Driving NK Cell Dysfunction

**Figure 4:**
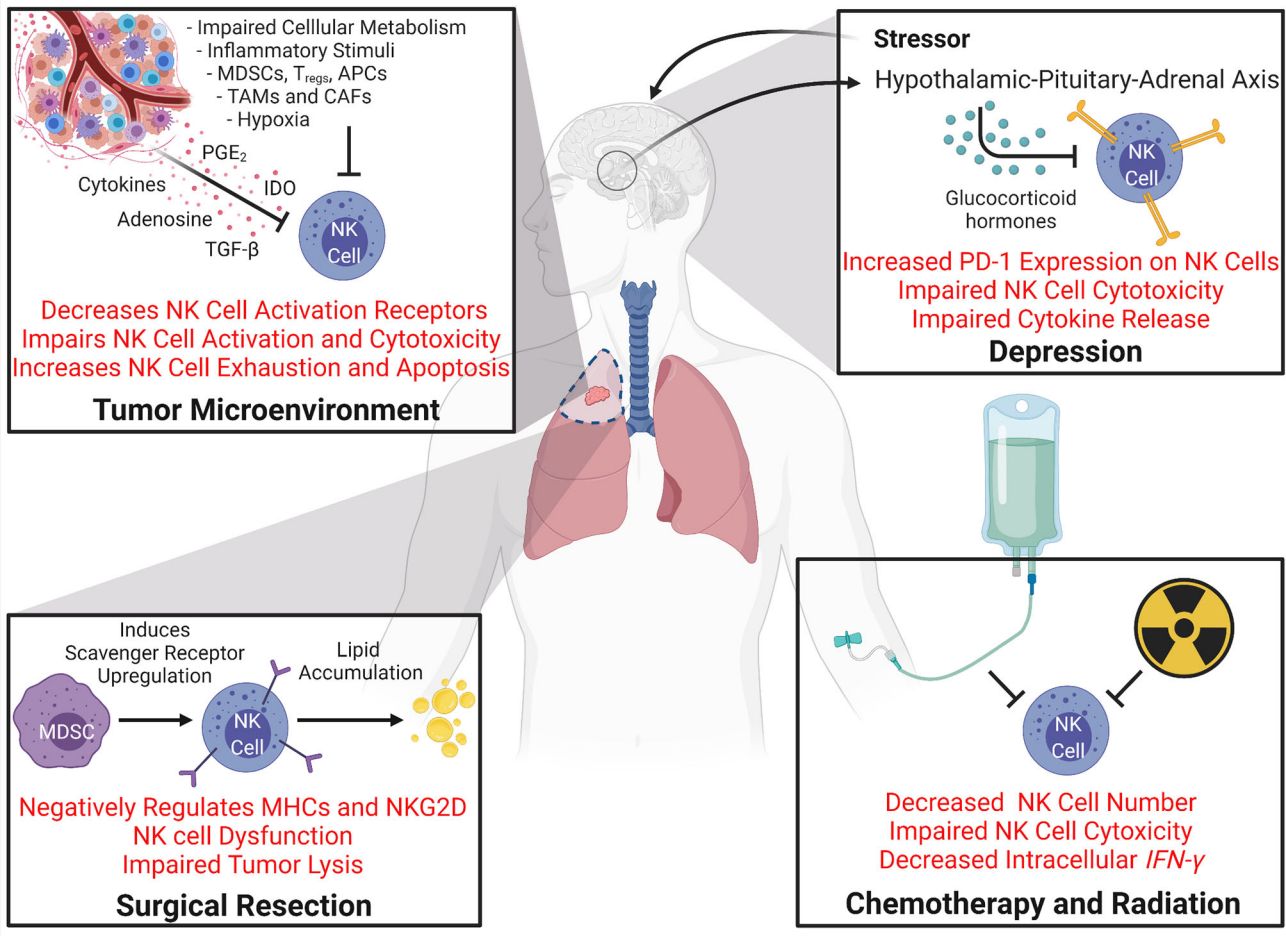
Mechanisms Driving NK Cell Dysfunction During Cancer. Many processes that occur during cancer and cancer therapy can cause dysfunction of NK cells. The tumor microenvironment itself creates a setting full of NK cell inhibitory mechanisms. Impaired cellular metabolism increased inflammatory stimuli, hypoxia, and the localized immunosuppressive cells all can promote NK cell deactivation and impair NK cytotoxicity. Secreted molecules like cytokines, adenosine, TGF-β, prostaglandin E2 (PGE2) (5), and Indoleamine 2,3-dioxygenase (IDO) in the tumor milieu also promote NK cell downregulation, exhaustion, and apoptosis [reviewed in 138)]. Secondary effects of cancer and cancer therapy, such as depression can also affect NK cell function. Stressors can activate glucocorticoid hormone production *via* the hypothalamic-pituitary-adrenal axis which can induce PD-1 expression on NK cells and impairs NK cell cytotoxicity and cytokine release. Cancer therapies such as chemotherapy, radiation, and surgical resection can all cause NK cell dysfunction. Both chemotherapy and radiation have been shown to decrease NK cell population and impair NK cell cytotoxicity and IFN*-γ* levels. Surgical resection and perioperative factors have been shown to impair NK cell function. For example, increases in immunosuppressive cell populations such as MDSCs induce scavenger receptor expression on NK cells which promotes lipid accumulation which negatively regulates NK cell receptors and results in NK cell dysfunction.

New and emerging studies clearly demonstrate a link between NK cell function and the success of many cancer immunotherapies. NK cells are either direct targets of the immunotherapeutics or are indirectly affected by cells upon which the immunotherapies act on, and positive responses to these immunotherapies are linked to having a functional NK cell population to initiate and prime the immune system for productive anti-tumor response. Thus, understanding mechanisms that can cause dysfunction of NK cells is important for developing and further improving immunotherapeutic strategies for treating cancer (**Figure 4**). Not only does NK cell dysfunction occur due to immunosuppressive environment progressively established during tumor development, but many of the first-line treatment options also negatively impact NK cell function. The following section will review mechanisms of dysfunction induced by cancer and responses to it.

### NK Cells and Cancer-Induced Dysfunction

NK cells clearly fill a seminal role in orchestrating the body’s immunological defenses, thus when the NK cell compartment becomes dysfunctional or damaged, serious health problems can ensue. In an eleven-year prospective cohort study investigating natural immunological host defenses in healthy individuals, participants with low peripheral blood lymphocyte cytotoxic activity had a significantly higher risk for cancer incidence relative to those with medium or high cytotoxic lymphocyte activity (139). In this study, select lifestyle factors such as maintaining a healthy body weight, consuming green vegetables, and not smoking made minor attributions to heightened cytotoxic activity (139). Cytotoxic activity may be used as biomarkers to identify new lifestyle-centered cancer interventions. NK cells are an important brake preventing initial stages of tumor growth, however, as tumors development progresses, NK cell antitumor forces gradually wane as tumor factors promote NK cell exhaustion (31).

Immune evasion is a hallmark of cancer. Tumors employ an arsenal of tactics to escape destruction – either by eluding immunosurveillance or disabling the immune response – several of which were previously touched upon in this review. Chronic exposure to inflammatory stimuli is a major factor driving NK cell dysfunction by compromising proliferative capacities and crippling effector functionality, ultimately resulting in pathogenesis. Tumor-associated NK cells exhibit an altered activation receptor repertoire and a diminished cytotoxic capacity compared to NK cells from healthy donors, supporting the assertion that the tumor microenvironment remodels the immune profile (33, 140–142). Tumors secrete cytokines that localize Tregs, myeloid-derived suppressor cells (MDSCs), tumor-associated macrophages, and cancer-associated fibroblasts – major components of the immune-suppressive network – to the tumor milieu (143, 144). These cells are a primary source of immunosuppressive cytokines that are responsible for the subdual of the anti-cancer immune response, importantly TGF-β. TGF-β signaling exerts direct effects over the downregulation of several NK cell activation receptors in an array of cancers (141, 145, 146). Recent studies report that TGF-β participates in additional immune-obstructive mechanisms, constructing stromal barriers that exclude lymphocytes from the tumor parenchyma, disrupting NK cell tumor-trafficking by negatively modulating the CX_3_CL1/CX_3_CR1 chemokine/chemokine receptor axis, curbing NK cell cytotoxicity in metastatic breast cancer by restricting NK cell metabolism and regulating leukemia cell susceptibility against NK cell targeting by down regulating expression of CD48 (147–151). It has also been reported that TGF-β signaling within the tumor microenvironment promotes the conversion of NK cells into intermediate type I innate lymphoid cells that are unable to control local tumor growth and metastasis, driving tumor evasion from the innate immune system (152). Elevated TGF-β was also found to upregulate FBP1 in KRAS-mutant lung cancer (31). FBP1-blunted glucose metabolism reduces NK cell viability and disarms the hold NK cells have over tumor initiation (31). Combination anti-PD-L1 and TGF-β agonist therapy is now being evaluated (153).

While some methods of immune evasion are more universally applied, cancers exploit a diverse battery of tumor-specific evasion methods as well. Production of soluble IL-2Rα (sIL-2R/sCD25) by Reed-Sternberg (RS) cells in classic Hodgkin lymphoma, binds IL-2, reducing its bioavailability for NK cell activation and proliferation; elevated serum sIL-2R levels are linked to more aggressive disease states and poorer clinical outcomes (154, 155). The estrogen pathway is also engaged in the management of the innate and adaptive immune system. Elevated levels of estrogens promote proteinase inhibitor 9 expression, which protects breast cancer cells from granzyme B-induced apoptosis *in vitro* (156). For a more detailed review of additional tumoral mechanisms shaping NK cell anti-tumor functions, see (144, 157, 158) .

The immunosuppressive tumor microenvironment is also a dominant force in cancer resistance to immunotherapy and checkpoint inhibitors. There is a growing body of evidence that identifies hypoxic stress as a mechanism by which tumors elude immune surveillance. Hypoxia in the tumor microenvironment has broad spectrum debilitating effects that are evident at every level of the anticancer response: impairing T-cell infiltration, blunting the cancer attack mounted by NK cells, attracting immunosuppressive Tregs, and promoting intratumoral heterogeneity (159, 160). Hypoxia-driven suppression of NK cell activity has a complex, multimodal mechanism of inhibition. Secretion of TGF-β and hypoxia-inducible factor-1α by tumors decreases the NKG2D activating receptor on NK cells and NKG2D ligand on tumor cells, respectively, tipping the scale in favor of NK cell inhibition (161). NK cell proliferation and cytotoxicity are further checked by adenosine A2A receptor-mediated signaling. The accumulation of extracellular adenosine in the tumor microenvironment by CD39 and CD73 ectonucleotidases is an additional method that protects tumors from the NK cell response (160). The effects of the tumor microenvironment on NK cell dysfunction are summarized in **Figure 4**.

### NK Cells and Surgery

Surgical resection of primary tumors and metastatic lymph nodes is often the first-line treatment of cancer. While surgical tumor debulking has immediately apparent benefits, the lingering adverse aftereffects of surgery present a concern for postoperative recovery, residual tumor control, and relapse-free survival. To this effect, a retrospective study on breast cancer, evaluating the mortality distribution for patients undergoing mastectomy versus untreated patients, found a bimodal death-specific hazard distribution in patients receiving mastectomies (162). In patients with underlying malignancies the immune system is already compromised. The postoperative stress response further weakens NK cell-led immunity, opening an immunological window of opportunity conducive to immune evasion, metastasis development, and accelerated residual tumor outgrowth, which provides a logical explanation for the observed double-peaked pattern (162). A host of perioperative factors – surgical trauma, anesthetics, analgesics, and blood transfusions – provoke stress-related factors, anti-inflammatory cytokines, and immunosuppressive cell populations that shape the postoperative immune climate. Tai et al. observed an expanded MDSC population in surgically-stressed mice (163). MDSCs induce scavenger receptor upregulation on NK cells which results in lipid accumulation. Postoperative lipid accumulation in NK cells negatively regulates the mouse MHC receptor repertoire – Ly49A, Ly49E/F, and Ly49G2 – and activating receptor NKG2D, resulting in NK cell dysfunction and impaired tumor lysis (164). Hypercoagulability is an intrinsic response to surgically-induced platelet activation. In this state, fibrin and platelets form peritumoral aggregates around tumor cell emboli, shielding tumor cells from NK cell-mediated extermination and promoting tumor metastasis (165). Surgical support efforts such as anesthetics, analgesics, and allogenic blood transfusion also have direct and indirect effects on immune effector cells, the hypothalamic-pituitary-adrenal (HPA) axis, and sympathetic nervous system. These effects can induce a stress response, contribute to postoperative immune system attenuation, poorer prognoses, and have been identified as risk factors for cancer recurrence (166–170). NK cells isolated from a small cohort of patients receiving transfusions exhibit a decline in NK cell-mediated lysis that is likely shaped by TGF-β and soluble HLA-type I and FasL (171). A number of studies report that NK cell impairment can persist for up to thirty days postop (163, 172–174). The mechanisms governing postoperative NK cell dysfunction, summarized in **Figure 4** are incompletely understood, however, further insight will aid in honing new clinical interventions.

### NK Cells and Chemo/Radiation

Chemotherapy and radiation are also conventional oncologic interventions. As targeted immunotherapies are gaining traction, there is increasing interest in utilizing these strategies secondary to initial tumor reduction using traditional chemotherapy and radiation techniques. Exploring the largely unstudied immunological consequences chemotherapy and radiation bear on the immune system is important to gauge the potential for success of these treatment pairings. The current body of literature examining the functionality of the immune system after chemotherapy and radiation largely focuses on the immune system as a whole. Following the first round of chemotherapy, the total lymphocyte population showed a significant reduction compared to baseline populations (175, 176). The survival outcome of these patients was dependent on the capacity for T cell populations to recover in the wake of chemotherapeutic-induced immunological changes (175, 176). In line with the observed effects of chemotherapy on lymphocytes, absolute levels of NK cells and intracellular IFN*-γ* levels were significantly higher prior to radiation or radiation and chemotherapy (177). Supporting this observation, a recent study using an established murine hepatic irradiation model, showed that hepatic irradiation decreased the number of liver resident NK cells and the effect correlated with hepatic irradiation dose (178). Liver resident NK populations did not recover by two months post irradiation and the irradiation prevented differentiation of precursor cells into liver resident NK cells, however adoptive transfer of activated NK cells could alleviate metastatic growth (178).

In patients with hematologic malignancies receiving haploidentical hematopoietic stem cell transplants, cyclophosphamide, a potent immunosuppressive agent, is commonly administered post-transplantation to eliminate alloreactive donor T lymphocytes and mitigate potential graft-*vs*-host disease (179–181). Russo et al. observed decreases in donor-derived NK cell counts following cyclophosphamide infusion, suggesting that highly proliferating graft NK cells are also targets of cyclophosphamide’s selective elimination, potentially attenuating the NK cell-mediated graft-*vs*-leukemia attack (182). Two weeks after cyclophosphamide infusion, a second, less mature and less cytotoxic population of donor NK cells begins to emerge, and full reconstitution of a mature NK cell compartment may not be complete for up to a year after transplant (182, 183). These studies highlight the need to consider the consequences of chemotherapeutics and radiation treatment on immune cell populations and immunotherapies and potential need for adoptive cell therapies. The effects of chemotherapy and radiation on NK cells is summarized in **Figure 4**.

### NK Cells and Depression

Depression is a common occurrence in cancer patients and has been highlighted as an important co-morbidity to understand (184). One study showed pooled mean prevalence of depression in cancer patients ranged from 8-24% (185) and another has shown the odds of being depressed are five times higher in cancer patients (186). Some cancers can release chemicals that are thought to cause depression and even certain cancer treatments, such as chemotherapy and corticosteroids, are associated with depression (187). Glucocorticoids are steroid hormones released following activation of the HPA axis and are key regulators of the innate and adaptive immune responses, including NK cell activity (188), forging a link between the neuroendocrine and immune systems (**Figure 4**). Interestingly, hyperactivity of the HPA axis is observed in patients with depression, resulting in excessive glucocorticoid release, impairment of NK cell cytotoxicity, and subsequent cancer progression (62). Glucocorticoids bind the ubiquitously expressed glucocorticoid receptor (GR), curbing immune-mediated inflammation *via* suppression of the cytolytic activity of and the production of pro-inflammatory cytokines by immune cells. A study by Yang et al. examining glucocorticoid release in response to psychological distress found that stress-induced glucocorticoids led to upregulation of the immunosuppressive factor *Tsc22d3*, resulting in repression of the dendritic cell-mediated type I IFN response required for the activation of adaptive anticancer surveillance efforts (189). Endogenous glucocorticoids are also associated with increased *de novo* PD-1 expression on NK cells. Glucocorticoids in combination with IL-15 and IL-18 selectively induce PD-1 expression on splenic NK cells, which negatively regulates the IFN*-γ* response of NK cells (187). These results establish the GR-PD-1 axis as a novel mechanism of neuroimmune regulation.

Glucocorticoids are often prescribed to palliate some of the side effects of chemotherapies and radiation, however, the data presented above suggests exogenous glucocorticoids may actually obstruct therapy-driven immune stimulation and control of tumor growth. Further research is required to modify current standard patient management strategies. In light of favorable data, pharmacologic and psychosocial therapies targeted to decrease glucocorticoid pathway activity may be considered as supplemental therapies to harness the full potential of immunotherapies and checkpoint inhibitors.

## Current Advances in NK Cell Therapies

**Figure 5:**
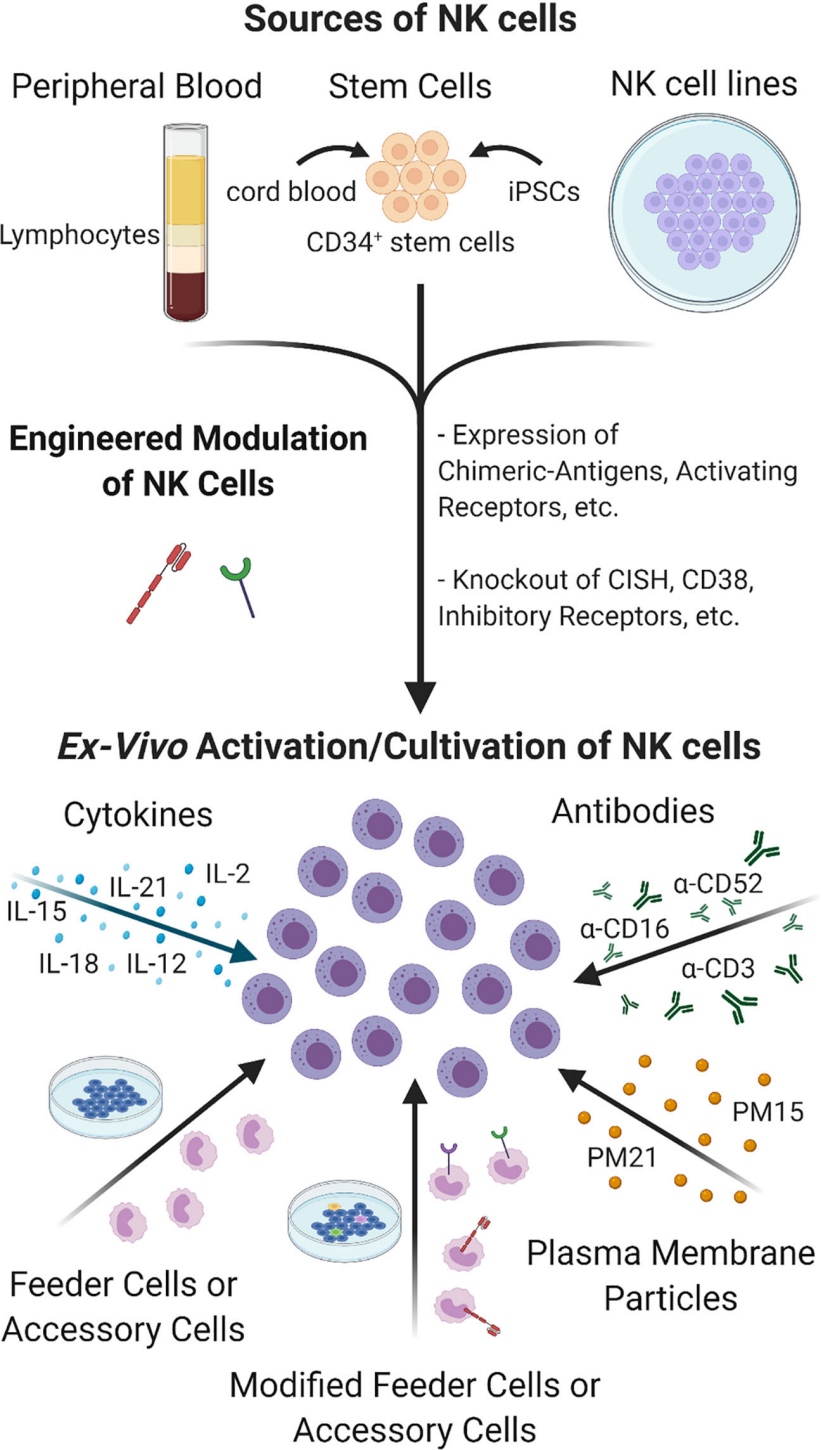
Sources and Cultivation of NK cells. NK cells can be extracted from peripheral blood or be differentiated from CD34^+^ stem cells sourced from cord blood, placenta or manufactured from iPSCs. Tumor-derived NK cell lines are also being developed to expand large numbers of NK cells *ex vivo*. NK cells can be engineered to express cytokines, natural or modified receptors, or transformed to knock out inhibitory receptors and other molecules to enhance their cultivation, targeting and activity under TME. Unmodified or genetically engineered NK cells can be further activated and/or expanded by culturing in the presence of cytokines or antibodies alone or in combination with co-culturing with feeder cells or accessory cells, which themselves can be modified for greater activation. Feeder cell-free NK expansion methods have also been developed such as using plasma membrane particles that provide robust expansion of highly cytotoxic NK cells comparable to feeder cell- based methods without the drawbacks and safety concerns.

A functional NK cell population is imperative to improving the efficacy and durability of cancer immunotherapies and combination treatments with adoptive NK cell therapies is an emerging strategy. NK cells comprise a minor portion of the circulating lymphocyte population. Devising protocols to selectively expand sufficiently large numbers of NK cells *ex vivo* for clinical infusion therapies has precluded NK cell-based therapies until recently, the recent advances are summarized in **Figure 5**. Co-culturing NK cells with K562 feeder cells engineered to express membrane bound 4-1BBL and IL-15 or IL-21 has proven to be an effective method for attaining robust NK cell expansion (190, 191). Preliminary data from a Phase I clinical trial evaluating the feasibility, safety, and dose-escalation response of high-dose infusion of haploidentical NK cells, expanded *ex vivo* using membrane bound IL-21 (mbIL21)-K562 feeder cells, in high-risk leukemia patients has generated encouraging data, with low observed rates of relapse, viral reactivation, graft-*vs*-host disease, and no dose-related toxicity (35). Updates from the Phase II extension of the study have continued to be positive, with one year relapse at 8% and two year progression-free survival at 66% in the 25 patients enrolled to date (192). Two additional Phase I studies are currently underway for patients with relapsed/refractory myeloid leukemias. With 13 patients treated thus far, 69% have achieved complete remission (193).

Despite taking precautions, concerns over the risk feeder cell-expanded NK cells carry for the potential infusion of tumor-derived feeder cells or tumorous material to patients have led to the exploration of feeder cell-free NK expansion methods. Oyer et al., pioneered a novel, feeder cell-free NK cell expansion method using plasma membrane particles (PM-particles) derived from K562 feeder cells genetically engineered to express 4-1BBL and mbIL21 or mbIL15 (194, 195). *Ex vivo* NK cell expansion and cytotoxicity levels using particles were comparable to levels achieved using feeder cell methods (195). Haploidentical PM21- NK cells are currently tested in Phase II clinical trials (NCT 04395092) as post-transplant relapse prevention in AML and myelodysplastic syndromes.

An alternative approach that avoids the use of feeder cells utilizes short preactivation with cytokines. The combined IL-12, IL-15, and IL-18 *in vitro* preactivation of NK cells does not lead to expansion of NK cell *in vitro* but is capable of provoking a durable memory-like IFN*-γ* response upon secondary stimulation (196). These memory-like NK cells, referred to as cytokine induced memory-like (CIML) NK cells, demonstrated heightened antileukemia responses that persisted for one month after infusion into patients, propelling four out of nine patients into complete remission (197, 198). There are currently seven ongoing clinical trials listed in the NCT database using CIML NK cells in combination treatments for AML and multiple myeloma. NK cell cytokine pre-activation may be incorporated into current *ex vivo* therapeutic NK cell manufacturing practices, allowing for NK cell memory to be harnessed and exploited to further amplify other immunotherapies.

The above mentioned methods utilize PBMCs as source of NK cells but other sources such as cord blood, placental or iPSCs derived stem cells have also been utilized as starting source for NK cells (199, 200) [reviewed in (201)]. Current trends in NK cell therapy are also focused on using genetic and non-genetic methods to improve NK cell expansion, cytotoxicity, targeting, homing, and to increase lifespan (189, 202) [reviewed in (203, 204)]. Among them, chimeric antigen receptor (CAR)-engineered NK cells have been a major emerging method for cancer therapy. For example, an ErbB2 (HER2)-specific CAR-NK is currently being used in a phase I clinical trial for treatment of glioblastoma patients (205). In a phase I and II clinical trial HLA-mismatched anti-CD19 CAR-NK cells derived from cord blood was administered to patients with B-Lymphoid malignancies and saw a 73% response (206) and now early phase I clinical trials are further investigating CD19 and CD22 CAR-NK cells in Refractory B-cell Lymphoma patients, NCT03690310 and NCT03692767. Recently an open label pilot study also began to evaluate the safety and feasibility of CAR-NK cells targeting NKG2D ligands in the treatment of metastatic solid tumors, NCT03415100. Many recent reviews on the subject have been published, see (207–211). For a more detailed review of the current state of NK cell *ex vivo* cultivation see (207) and the use of adoptive NK cell immunotherapies, see (212).

## Conclusion

The immune system’s carefully orchestrated anti-tumor response draws its power from the concerted contributions of the innate and adaptive immune arms. The NK cell kicks off the first leg of the immunological response: patients lacking a robust NK cell compartment are unable to mount strong killing of malignant cells and fail to harness the full therapeutic effects of immunotherapies.

This presents a strong argument in favor of employing adoptive NK cell transfer prior to or concomitantly with immunotherapies to jumpstart the immune response. Precursory adoptive NK cell transfer may reconstitute the NK cell compartment, providing the necessary priming of the immune system for optimal activity of subsequent effector populations and maximizing therapeutic efficacy.

Immunotherapy is a promising new frontier in cancer treatment. Great strides are being made in the breadth and availability of cancer therapeutics, however, variability in patient responses remains a chronic barrier to further success. Cytotoxic T lymphocytes are a major focus of immunotherapies, however, increasing reports suggest other effector populations are critical to a positive therapeutic response and should be given equal attention in study design. Dually tasked with effector and regulatory functions, the NK cell is the linchpin of the complex immune response: directly responsible for lysis of tumor cells through ADCC and the clearance of MHC-compromised cells in the primary immune response and priming of the tumor microenvironment through PD-L1 induction on tumors and recruitment of DCs and subsequently T cells for the secondary adaptive immune response. Future immunotherapy treatment protocols should consider deeply the synergy of the innate and adaptive immune system in order to further improve cancer treatment and long-term tumor control.

## Author Contributions

KS: Conceptualization, literature research, writing - review & editing. TC-P: Literature research, writing – figure preparation, review & editing. AC: Conceptualization, literature research, funding acquisition, project administration, resources, supervision, writing - review & editing. All authors contributed to the article and approved the submitted version.

## Funding

We would like to thank the Florida Department of Health, James and Ester King Biomedical Research Grant program (grant# 9JK04 to AJC) and The Guillot-Henley Family AML Research Fund in loving memory of William L. Guillot for financial support.

## Conflict of Interest

AC has intellectual property licensed to and holds ownership interest in as well as consults for Kiadis Pharma. AC also received research support from Kiadis Pharma.

The remaining authors declare that the research was conducted in the absence of any commercial or financial relationships that could be construed as a potential conflict of interest.

## Glossary

**Table:**

2B4	CD244 natural killer cell receptor 2B4
4-1BBL	4-1BB ligand
ADCC	antibody-dependent cell-mediated cytotoxicity
AML	acute myeloid leukemia
AMP	adenosine monophosphate
APCs	antigen-presenting cells
CCL3	C-C motif chemokine ligand 3
CCL5	C-C motif chemokine ligand 5
CCR5	C-C motif chemokine receptor 5
CD16	Fc fragment of IgG receptor III&alpha
CD25	IL2R&alpha interleukin 2 receptor subunit alpha
CD39	ENTPD1 ectonucleoside triphosphate diphosphohydrolase 1
CD54	ICAM1 intercellular adhesion molecule 1
CD73	NT5E 5&rsquo;-nucleotidase ecto
CD107a	LAMP1 lysosomal associated membrane protein 1
CD137	4-1BB/TNFRSF9 TNF receptor superfamily member 9
cDC1	conventional type 1 dendritic cells
cGAMP	cyclic guanosine monophosphate-adenosine monophosphate
cGAS	cyclic guanosine monophosphate-adenosine monophosphate synthase
CIML NK cells	cytokine-induced memory-like Natural Killer cells
CTLA-4	cytotoxic T-lymphocyte associated protein 4
CX_3_CL1	C-X3-C motif chemokine ligand 1
CX_3_CR1	C-X3-C motif chemokine receptor 1
CXCL9	C-X-C motif chemokine ligand 9
CXCL10	C-X-C motif chemokine ligand 10
DCs	dendritic cells
DNAM-1	CD226 molecule
ERK	extracellular signal-regulated kinase
FasL	Fas ligand
FBP1	fructose-bisphosphatase 1
FLT3LG	fms related receptor tyrosine kinase 3 ligand
Foxp3	forkhead box P3
GMP	cyclic guanosine monophosphate
HER2/NEU	erb-b2 receptor tyrosine kinase 2
HLA	human leukocyte antigen
HLA-E	major histocompatibility complex
class I	E
HPA	hypothalamic-pituitary-adrenal
HVJ-E	hemagglutinating virus of Japan-Envelope
IFN	interferon
IRF3	interferon regulatory factor 3
iPSCs	induced pluripotent stem cells
IRF3	interferon regulatory factor 3
KIR	killer cell immunoglobulin like receptor
mbIL21	membrane bound IL-21
MDSCs	myeloid-derived suppressor cells
MEK	mitogen-activated protein kinase
MHC	major histocompatibility complex
MSI-H/dMMR	microsatellite instability-high or mismatch repair deficient
NCRs	natural cytotoxicity receptors
NDV	Newcastle Disease Virus
NF-κβ	nuclear factor κβ
NK cells	Natural Killer cells
NKG2A	KLRC1 killer cell lectin like receptor C1
NKG2D	KLRK1 killer cell lectin like receptor K1
NKp30	NCR3 natural cytotoxicity triggering receptor 3
NSCLC	non-small cell lung cancer
OV	oncolytic virus
PBMCs	peripheral blood mononuclear cells
PD-1	PDCD1 programmed cell death 1
PD-L1	CD274/programmed cell death 1 ligand 1
PM21-particles	plasma membrane particles
RAE-1	retinoic acid early inducible 1
S100	ADU-S100
STING	stimulation of interferon genes
TCR	T cell receptor
TGF-β	transforming growth factor beta
TIGIT	T cell immunoreceptor with Ig and ITIM domains
TIM-3	T cell immunoglobulin and mucin domain containing 4
TNF-α	tumor necrosis factor alpha
Tregs	regulatory T cells
XCL1	X-C motif chemokine ligand 1.
